# A New Protocol to Treat Abdominal Subcutaneous Fat Combining Microwaves and Flat magnetic stimulation

**DOI:** 10.3390/bioengineering9050182

**Published:** 2022-04-21

**Authors:** Steven Paul Nisticò, Paolo Bonan, Federica Coli, Alice Verdelli, Irene Fusco, Francesco Gratteri, Claudia Sicilia, Carmen Cantisani, Giovanni Pellacani, Luigi Bennardo, Giovanni Cannarozzo

**Affiliations:** 1 Department of Health Sciences, Magna Graecia University, 88100 Catanzaro, Italy; luigibennardo10@gmail.com; 2 Laser Cutaneous Cosmetic & Plastic Surgery Unit, Villa Donatello Clinic, 50121 Florence, Italy; dr.pbonan@gmail.com (P.B.); coli.federica.vd@gmail.com (F.C.); alice.verdelli83@gmail.com (A.V.); 3 Department of Pharmacology, University of Florence, 50121 Florence, Italy; irene.fusco@unifi.it; 4 Dermatology Unit, Department of Medicine (DIMED), University of Padova, 35100 Padua, Italy; francescogratteri92@icloud.com; 5 Plastic Surgery Unit, Department of Human Pathology, University of Messina, 98121 Messina, Italy; claudiasiciliakr@gmail.com; 6 Department of Dermatology, Policlinico Umberto I, Sapienza University of Rome, 00100 Rome, Italy; carmencantisanister@gmail.com (C.C.); pellacani.giovanni@gmail.com (G.P.); drcannarozzo@gmail.com (G.C.)

**Keywords:** abdominal subcutaneous fat, microwaves for body shaping, flat magnetic stimulation

## Abstract

Background: A healthy lifestyle is not always able to improve the abdomen’s appearance, especially in those patients who have undergone sudden weight changes. Objective: We aimed at evaluating the efficacy of combined microwaves and flat magnetic stimulation (FMS) to treat abdominal localized adiposity and laxity. Methods: Twenty-five patients were subjected to two treatment sessions per month on the abdominal area with microwaves. FMS was also performed twice per week, with a minimum of two days between each session for two months. The technology uses three types of different protocols: massage, muscle definition (shaping), and muscular strengthening. Measurements, including body mass index (BMI) and waist, and abdominal ultrasound were performed at baseline and three months after the last treatment session. Blood examinations were performed, and a 5-Likert scale questionnaire was used to assess patient satisfaction. Results: At follow-up, three months after the last treatment, the mean waist circumference (WC) was significantly reduced, and skin laxity improved in all patients (*p* < 0.001). A significant improvement in abdominal muscle tissue thickness was also shown in all abdominal areas, and the thickness of the adipose tissue evaluated by ultrasound was reduced. Conclusions: This study proves that the combination of microwaves and FMS treatment is secure and efficient for treating abdominal subcutaneous fat and skin laxity.

## 1. Introduction

Localized abdominal subcutaneous fat deposits and tissue laxity are among the most common aesthetic problems in both sexes, with a growing demand for aesthetic treatments [1]. Many procedures, including surgery, lasers, and medical approaches, have been developed with satisfactory results [2,3,4,5]. Surgery remains the first choice treatment, but its invasive nature has led to the demand for less invasive therapies [2]. Among the new laser technologies, microwaves recently showed a promising role in body shaping [6,7], improving the abdominal subcutaneous adipose tissue through a non-invasive laser system, without side effects and the need for extended recovery times. Lifestyle has a significant role in maintaining the obtained results. A correct diet based on the consumption of fiber and protein rather than sugar, salt, and carbohydrates and regular physical activity are essential [8]. However, a healthy lifestyle is not always able to improve the abdomen’s appearance, especially in those patients who have undergone sudden weight changes. 

Sometimes patients with weak abdominal muscles are dissatisfied with their outward appearance after abdominal fat removal because there may be swelling and flaccidity around the abdominal wall. Therefore, the removal of excess fat does not solve the problem of muscle flaccidity developed by reducing muscle and aponeurotic tension and increasing intra-abdominal pressure. An additional obstacle is that surgical and non-invasive body contouring procedures require patients with well-defined bulges for effective and safe treatment. Furthermore, none of these methods affect the underlying muscles, not giving a toned abdominal appearance. Therefore, patients with a lower body mass index (BMI) are not eligible for this procedure. Recently, high-intensity focused electromagnetic (HI-FEM) field technology has shown promising results in body shaping. The application of HI-FEM diverts the peripheral and central nervous system by directly stimulating the motor neurons that innervate the muscles, thus allowing muscle contraction, with a direct action on abdominal laxity and flaccidity. Based on our experience and the efficacy of microwaves on subcutaneous fat and skin laxity, [6,7] and according to a new electromagnetic stimulation technology, flat magnetic stimulation (FMS), [9] we aimed at evaluating the efficacy of combined microwaves and FMS to treat abdominal localized adiposity and laxity, carrying out a synergic action on the connective and muscle tissue.

## 2. Materials and Methods

Patients with abdominal localized subcutaneous fats and laxity of both sexes, aged > 18 years, were enrolled. Exclusion criteria included BMI >30, pregnancy or breastfeeding, menstruation, present or past oncological diseases, systemic infections and immunosuppression, cardiovascular diseases, implanted electronic devices, metal implants, cardiac pacemakers, and other medical conditions contraindicating the use of the electromagnetic field. Patients who had undergone abdominal surgery, laser treatment, or topical/injective drug therapies in the previous six months were also excluded.

At baseline, demographic data, anthropometric measurements [BMI, waist circumference (WC), skin laxity (0–3 scale; 0 = no laxity, 1 = mild, 2 = moderate, 3 = severe)] and blood examinations were collected. Patients preserved their quotidian diet and physical activities without any variations until study completion. Patients did not take any supplemental fat-reducing elements and they did not receive other body treatments during the treatment period.

The study was performed according to the Declaration of Helsinki, and all the participants signed informed consent.

### 2.1. Experimental Protocol

Each patient was subjected to two treatment sessions per month on the abdominal area with the ONDA Coolwaves^®^ system (DEKA, Florence, Italy). The ONDA Coolwaves generates waves at 2.45 GHz, producing localized, controlled heat absorbed by fat through a biophysical process called “dielectric heating” [6]. According to the treated area, sex, degree of laxity, and fat deposits, standardized parameters were chosen. FMS with the Schwarzy^®^ system (DEKA, Florence, Italy) [9] was also performed twice per week, with a minimum of two days between each session for two months. The FMS generates electromagnetic pulses with an intensity of up to 2.5 Tesla (T). The pulse duration was 250 µs ± 20%, and the pulse repetition frequency was 1–150 Hz. The technology uses three types of different protocols: massage, muscle definition (shaping), and muscular strengthening; it is customizable based on muscle condition, adapting to the needs of each type of patient. During the treatment, the applicator of the elliptical device was positioned above the navel of the patient who was placed in the supine position. At the start of the treatment, the position of the applicator was adjusted to ensure evenly distributed contractions and was fixed to avoid its possible movement during the treatment session. Depending on the type of patient, the treatment lasted from 20 to 45 min, and the muscles involved were the internal/external obliques and the rectus abdominis. The treatment protocol is reported in Table 1.

### 2.2. Objective and Patients’ Assessments 

Measurements, including BMI and waist, and abdominal ultrasound, were performed at baseline and three months after the last treatment session. 

The objective evaluation involved clinical photography and three-dimensional (3D) optical skin surface measurement (QuantifiCare S.A., Valbonne, France) [10]. Three blinded assessors evaluated randomized digital photographs for recognition. Subcutaneous fat, defined as the depth from the skin to the abdominal muscles, was measured by ultrasound and reported in millimeters (mm) (Toshiba Aplio i600, Vimercate, Italy) [11]. Four abdominal sites (upper abdomen, lower abdomen, lateral abdomen, rectus abdominis diastasis) were examined at the same distance from the same reference point.

Blood examination, including total cholesterol, complete blood count, low-density lipoprotein (LDL) cholesterol triglycerides, creatine kinase, and transaminases, was performed immediately before starting the treatment and three months after the last treatment.

A 5-Likert scale questionnaire was used to assess patient satisfaction after the last treatment and at the 3-month follow-up.

### 2.3. Statistical Analyses

The Wilcoxon signed-rank test was performed. Data were presented as means ± standard deviation (SD). A *p*-value < 0.05 was set as a cut-off for statistical significance.

## 3. Results

In total, 25 patients (20 females, 5 males) who wanted a more aesthetically pleasing abdomen voluntarily participated in this research. The patients’ mean age (±SD) ranged from 25 to 59 years with an average of 33 ± 7.2 years and a BMI of 24.8 ± 3.3 kg/m^2^ (range, 23–29 kg/m^2^).

### 3.1. Objective Measurements

At baseline, mean WC was 98.2 ± 7.4 cm (range, 77.2–113 cm). Moderate skin laxity was demonstrated in 56% (*n* = 14) of the patients, while 44% (*n* = 11) of the volunteers showed slight laxity. The median abdominal muscle tissue thicknesses evaluated by ultrasound were the following: 9.5 ± 3 mm for the upper abdomen, 10.3 ± 2.4 mm for the lower abdomen, 10.1 ± 2.0 mm for the lateral abdomen, 24.1 ± 4.1 mm for rectus abdominis diastasis.

**Figure 1:**
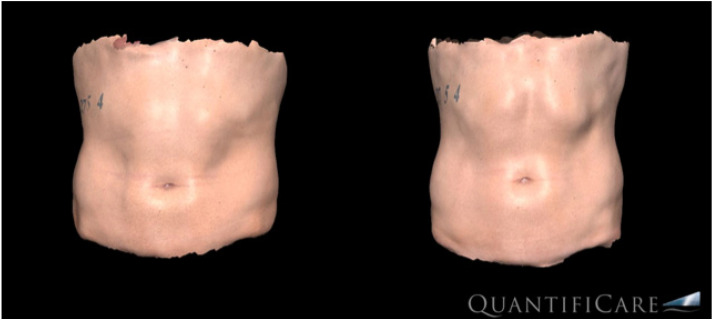
Three-dimensional (3D-Quantificare) optical abdominal skin surface image of a male patient before (**left**) and three months after the last treatment (**right**).

**Figure 2:**
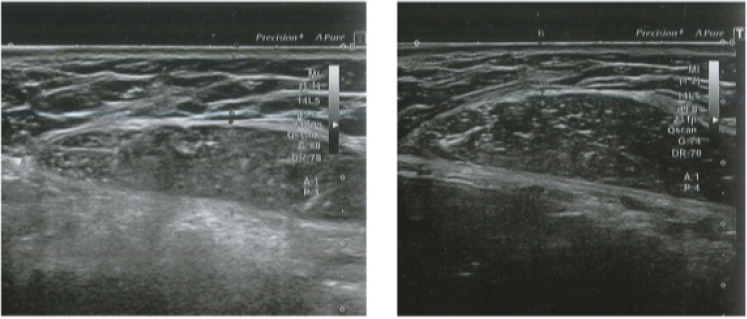
Abdominal echography of a male patient before (**left**) and three months after (**right**) the last treatment. Visible fat reduction and improvement of rectus abdominis muscle thickness in the treated area are observed.

**Figure 3:**
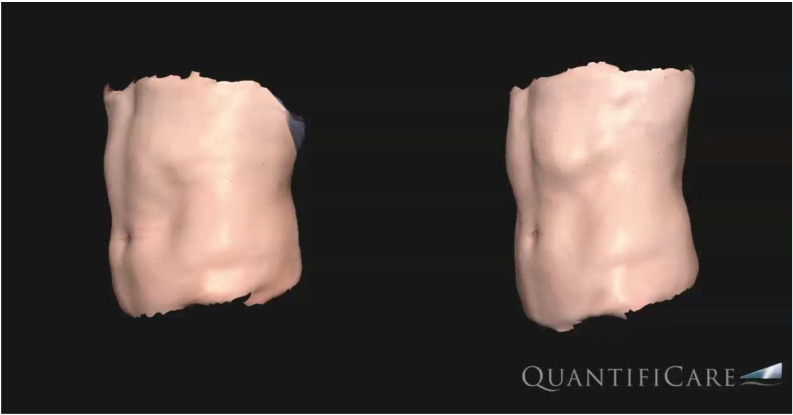
Three-dimensional (3D-Quantificare) optical abdominal skin surface image of a male patient before (**left**) and three months after the last treatment (**right**).

At follow-up, three months after the last treatment, the mean WC was 94.0 ± 6.6 cm with an average loss of 4.2 cm (*p* < 0.001). Skin laxity improved in all patients (*p* < 0.001), with 40% (*n* = 10) patients showing no laxity and 60% (*n* = 15) showing mild laxity three months after the last treatment. (Figure 1, Figure 2 and Figure 3). A significant improvement in abdominal muscle tissue thickness was also shown in the upper abdomen (12.0 ± 1.1mm, *p* < 0.001), lower abdomen (14.1 ± 2.1 mm, *p* < 0.001), lateral abdomen (13.2 ± 3.3 mm, *p* < 0.001), and rectus abdominis diastasis (22.1 ± 3.8 mm, *p* < 0.001). The thickness of the adipose tissue, evaluated by ultrasound, changed from 9.8 ± 2.4 mm to 8.0 ± 2.1 mm (Table 2, Figure 2).

No significant statistical BMI modifications were found at follow-up.

### 3.2. Blood Examination

At baseline, total cholesterol was 171.9 ± 40.7 (normal value < 200 mg/dL), LDL cholesterol was 105.8 ± 8.3 (normal value < 115 mg/dL), triglycerides were 116.2 ± 15.4 (normal value < 150 mg/dL), creatine kinase was 150.4 ± 28.2 (normal value < 171 U/L), aspartate aminotransferase (GOT) was 30 ± 4.0 (normal value < 34 U/L), alanine aminotransferase (GPT) was 37 ± 8.2 (normal value < 49 U/L), and gamma-glutamyl transferase (GGT) was 33.3 ± 8.0 (normal value < 73 U/L).

Blood values did not show significant changes at follow-up evaluations, particularly in creatine kinase and cholesterol values.

### 3.3. Patients’ Assessments and Side Effects

The treatment was well tolerated. According to the 5-Likert scale questionnaire, a median 3.0 ± 1.0 index was obtained after the last treatment session.

No side effects were reported after Onda Coolwaves^®^ treatment except for transient erythema in 18 patients (72%). The only side effect associated with the Schwarzy^®^ system was muscle soreness according to almost all patients (92%, *n* = 23), which was solved after 48 h.

## 4. Discussion

Many non-invasive techniques for reducing subcutaneous adipose tissue and skin laxity have been developed in recent years, with growing demand [2,3]. Each procedure can stimulate necrosis or apoptosis of adipose tissue with different mechanisms [12]. Improvement of the muscle mass under the subcutaneous tissue is also requested. Our study proposed a combined treatment with microwaves and FMS, remodeling both the subcutaneous and the muscle tissue, with satisfactory results.

Microwaves refer to electromagnetic waves at 2.5 kHz, which produce thermal energy by increasing the temperature of the adipose tissue and thereby stimulating the adipocyte apoptotic pathway [13,14]. Microwaves seem to create irreversible cytoplasmic and membrane damage to adipocytes [6,7]. Histopathologic and ultrasound examinations on subcutaneous adipose tissue samples of Vietnamese pigs demonstrated micro-vesiculation in the adipocytes, leading to necrosis and the activation of monocytes/macrophages on residual adipocytes components [14]. Concurrently, controlled hyperthermia determined reduction and fragmentation of interlobular collagen fibers in the septa, thus contributing to the remodeling of the subcutaneous tissue. Microwaves can directly heat collagen septa, causing the dissolution of deeper collagen fibers and activating fibroblasts, improving skin texture [15].

Clinical studies confirmed the efficacy of microwaves on body contouring [13]. Our workgroup showed a median abdominal circumference reduction of 3.90 cm (range 7–1.5 cm) in a cohort of 12 volunteers with localized abdominal adiposities after four sessions of ONDA Coolwaves^®^. Moreover, a significant improvement was also obtained in 15 postpartum females with abdomen laxity and striae distensae (mean circumferences reduction: 3.6 ± 1.2 cm) with combined microwaves and fractional micro ablative CO2 laser treatment, with the repositioning of the umbilicus in most cases. Microwaves specifically act on the subcutaneous fat layer, but they cannot strengthen the muscular foundations. To date, the only way to potentiate the core is to adopt a physical training protocol. Recently, HI-FEM devices have adopted magnetic field technology for toning and strengthening muscles [16]. HI-FEM consists of low-frequency wavelengths (3–40 Hz), which transport low energy. The available devices deliver pulses in a frequency that produces supramaximal contractions not achievable voluntarily. Electromagnetic stimulation acts directly on motor neurons, inducing involuntary contraction, while the surrounding tissue does not absorb the energy, and no thermal effects have been reported [17]. Muscle tissue is constricted to adapt to supramaximal contractions and responds with an intense remodeling of its internal structure, increasing muscle density and volume, thus leading to better muscle definition and tone. In addition, the body is subjected to a stressful condition, with the need for more energy to supply the contractions obtained through intensive lipolysis and the degradation of lipids into free fatty acids stored within adipocytes. Therefore, a secondary effect of HI-FEM includes subcutaneous tissue remodeling by lipolysis of adipocytes [16,17,18]. 

Previous studies demonstrated the role of HI-FEM in body shaping [16,17,18,19]. In work by Jacob et al. [16] on 41 patients, a median 5.87 ± 3.64 cm waist circumference reduction was reported after HI-FEM treatment, with increased muscle mass and fat thickness reduction. Moreover, the same group showed a reduction in the fat layer and width of abdominal separation in 10 postpartum women after HI-FEM treatment, while the thickness of abdominal muscle increased [19]. 

Histologically, Duncan et al. [20] showed significant histopathologic structural muscle changes in three Yorkshire pigs treated with HI-FEM with a combination of fiber hypertrophy and hyperplasia. Weiss et al. [21] demonstrated adipocyte volume decreased and fat apoptosis, with shape alterations and pyknotic nuclei, in the abdomen specimen of white pigs treated with radiofrequency (RF) and HI-FEM. Goldberg et al. [22] analyzed four human abdominal subcutaneous tissue treated with RF and HI-FEM. After treatment, the adipocytes underwent nuclear and shape changes (they were flattened and smaller, together with pycnotic nuclei with condensed nuclear chromatin) with consequent fat reduction. Overall, these studies seem to confirm the action of HI-FEM, alone or in combination with RF, on muscle and adipose tissue. However, only a few cases have analyzed human samples. More studies are needed to confirm the preliminary data and better understand which mechanisms are involved in muscle remodeling in humans. According to an instrumental investigation, e.g., computed tomography (CT), ultrasound, and magnetic resonance imaging (MRI), muscle growth, fat reduction, and abdominal separation reduction after HI-FEM treatment seem to be confirmed [18,19,23,24].

Among HI-FEM options, FMS is a new electromagnetic technology emitted by the Schwarzy^®^ system, generating electromagnetic pulses with an intensity of up to 2.5 Tesla (T).9 FMS technology acts either on the trophism of muscle mass or on reducing fat, inducing intense muscle contractions (20.000 per session). The innovation of the device is represented by its flat-type emission, which allows a more homogeneous distribution of the intensity that directly reaches the tissues in-depth, without any damage to the skin. The Schwarzy protocols have been programmed with active phases alternating with rest phases, allowing optimal muscle recovery of the patient and avoiding the onset of lactacidosis. Three pre-set programs are available, aerobic, shaping, and strength, which can be adapted according to the patient’s needs. In a preliminary study by Mezzana et al. [9] on 20 patients treated with FMS, a significant waist circumference reduction was observed in all patients and was retained for 3–6 months [9]. No other clinical studies on FMS are currently available. Our work shows data on combined treatment with microwaves and FMS for the first time. Our data show both a decrease in WC and improvement in patients’ abdominal muscle tissue thickness at 3-month follow-up, with the effect lasting months after completing the procedure. Treatment repetition once per year it is recommended to maintain the beneficial effects. The system produces a connective matrix remodeling of adipose tissue with successive modification of the microenvironment that regulates adipocytes metabolism. Therefore, the homeostatic balance between connective interstitium and adipocytes, responsible for the vitality of the adipose tissue, is altered, inducing metabolic modifications due to thermal stress in the adipocytes themselves, which are stimulated to release several lipids into the environment that surrounds them in quantities much higher than their physiological capacities [25].

Moreover, microwaves could result in adipocytes in response to the functional surcharge in the transporting mechanisms through membranes of the peripheral cytoplasm. Consequently, the localized dielectric heating could cause the rearrangement of adipocyte cytoplasm and irreversible damage to the cell membrane. These processes activate macrophages, responsible for the removal of adipocytes, thus resulting in subdermal fatty tissue reduction and circumference reduction. The treatment also releases proinflammatory mediators, such as (TNF)α, nitric oxide synthase, interleukin (IL)-6, and (IL)-1β. Most macrophages have a close relationship with adipocytes that have started the necrotic process, constituting characteristic “crown-like structures” [26]: the function of these structures is the removal of the excess of free fatty acids and lipid droplets free in the interstitial tissue, together with the residuals of dead adipocytes. These results confirm the treatment’s efficacy and safety, with patient satisfaction. Clinical improvements were confirmed by ultrasound, which demonstrated a strengthening of muscle tissue and a reduction in the adipocyte layer. No side effects were reported.

### Study Limitations

The limitations of the study are represented by the limited number of patients, the absence of a control group, the absence of a photographic evaluation of the effect of the intervention in the long term after the evaluated follow-up, and patient’s age and BMI, which can change the thickness of both the superficial and deep fatty layers of the anterior abdominal wall, thus influencing the plan and implementation of cosmetic procedures [27]. Therefore, further studies with a higher number of subjects, the presence of a control group, and an extended post-treatment period will be considered in order to confirm our preliminary results.

## 5. Conclusions

Attention and interest in the general population are growing to prevent skin aging and laxity [28,29]. This study proves that the combination of microwaves and FMS treatment is secure and efficient for treating abdominal subcutaneous fat and skin laxity. Acting both on muscle cells [9,30] and adipocytes [6,7], the treatment could improve body shape without being invasive.

## Figures and Tables

**Table 1:** Study Protocol.

1st Month	First Day of the Week	Third Day of the Week
1st week	Onda + Schwarzy (Massage)	Schwarzy (Massage)
2nd week	Schwarzy (Massage)	Schwarzy (Massage)
3th week	Schwarzy (Shaping/Strenght)	Schwarzy (Shaping/Strenght)
4th week	Schwarzy (Shaping/Strenght)	Schwarzy (Shaping/Strenght)
**2nd month**	**First day of the week**	
1st week	Onda + Schwarzy (Massage)	Schwarzy (Massage)
2nd week	Schwarzy (Massage)	
3th week	Schwarzy (Shaping/Strenght)	
4th week	Schwarzy (Shaping/Strenght)	

**Table 2:** Mean values of study parameters at baseline and 3-month follow-up.

	Baseline	3-Month Follow-Up
Mean WC (cm)	98.2 ± 7.4	94.0 ± 6.6
Abdominal muscle tissue thicknesses: upper abdomen (mm)	9.5 ± 3.0	12.0 ± 1.1
Abdominal muscle tissue thicknesses: lower abdomen (mm)	10.3 ± 2.4	14.1 ± 2.1
Abdominal muscle tissue thicknesses: lateral abdomen (mm)	10.1 ± 2.0	13.2 ± 3.3
Rectus abdominis diastasis (mm)	24.1 ± 4.1	22.1 ± 3.8
Thickness of the adipose tissue (mm)	9.8 ± 2.4	8.0 ± 2.1

## Data Availability

Data available upon reasonable request from the corresponding author.
